# Structure determination needs to go viral

**DOI:** 10.1007/s00726-023-03374-2

**Published:** 2024-01-29

**Authors:** Matheus de Bastos Balbe e Gutierres, Conrado Pedebos, Paula Bacaicoa-Caruso, Rodrigo Ligabue-Braun

**Affiliations:** 1 Programa de Pós-Graduação em Biociências (PPGBio), Universidade Federal de Ciências da Saúde de Porto Alegre - UFCSPA, Porto Alegre, Rio Grande do Sul Brazil; 2 Departamento de Farmacociências, Universidade Federal de Ciências da Saúde de Porto Alegre - UFCSPA, Porto Alegre, Rio Grande do Sul Brazil

**Keywords:** Virus, PDB, X-ray, NMR, Cryo-EM, Pandemic

## Abstract

Viral diseases are expected to cause new epidemics in the future, therefore, it is essential to assess how viral diversity is represented in terms of deposited protein structures. Here, data were collected from the Protein Data Bank to screen the available structures of viruses of interest to WHO. Excluding SARS-CoV-2 and HIV-1, less than 50 structures were found per year, indicating a lack of diversity. Efforts to determine viral structures are needed to increase preparedness for future public health challenges.

**Supplementary Information:**

The online version contains supplementary material available at 10.1007/s00726-023-03374-2.

## Introduction

The emergence of SARS-CoV-2 as the causative agent of the COVID-19 pandemic brought focus back to the emergence and re-emergence of infectious diseases as global health threats, especially those caused by viruses (Ciotti et al. 2020; da Silva et al. 2022). There is no established number of human-infecting virus species (and even the term ‘species’ might be inadequate), but a current estimate is of at least 219 virus species, under 23 families (Woolhouse et al. 2012). Despite these figures being estimates, data modeling and extrapolation propose from 513 novel viruses to 827,000 viruses as potentially human-infecting, from a universe of 1.67 million unknown viruses (Chatterjee et al. 2021). At least two thirds of these are of zoonotic origin, reinforcing the chance of transmission from farm or wild animals to humans (Pandit et al. 2022). In addition to that, half the viruses that can infect humans are also transmissible among humans, and half of those are able to generate more than one secondary case after infection (i.e. *R*_0_ > 1) (Woolhouse et al. 2012).

The potential risk of emerging infections led the World Health Organization (WHO) to prioritize some diseases for which there is epidemic potential and/or there are no or insufficient countermeasures (WHO 2023). The WHO priority list encompasses COVID-19, Crimean-Congo hemorrhagic fever, Ebola virus disease and Marburg virus disease, Lassa fever, Middle East respiratory syndrome coronavirus (MERS-CoV) and Severe Acute Respiratory Syndrome (SARS), Nipah and henipaviral diseases, Rift Valley fever, Zika, and “Disease X”. WHO defines Disease X as “the knowledge that a serious international epidemic could be caused by a pathogen currently unknown to cause human disease”, for which preparedness for other diseases could also be relevant (Chatterjee et al. 2021; WHO 2023).

The availability of protein structures is one of the basic requirements for rational drug design (Hol 1986; Sliwoski et al. 2013), especially the structure-based drug design route (Batool et al. 2019). In this strategy, the molecular target (generally a protein) is inspected in terms of its structure, providing stereoelectronic insights for the development of drug candidates (Mandal et al. 2009; Mavromoustakos et al. 2011). To obtain a protein structure experimentally (a process known as structure determination), an analytical technique is employed to solve three-dimensional atomic coordinates. These commonly include X-ray crystallography, NMR spectroscopy, and cryo-electron microscopy (Cryo-EM) (Stollar and Smith 2020).

In the case of SARS-CoV-2, there was an unprecedented output of resolved viral protein structures in a very short period of time (Lynch et al. 2021). A similar effort has only been seen for HIV, albeit on a much smaller scale in a much longer period of time (Engelman and Cherepanov 2012). Thus, considering the viral abundance (and its associated risks) in one hand, and the need for protein structure determination in the drug development pipelines in the other, the aim of this study was to assess how well represented is the viral diversity in terms of its protein structures as deposited in the RCSB Protein Data Bank. We were able to confirm a clear skew towards SARS-CoV-2 protein structures, followed by HIV, but at a very different speed of deposition. Other priority-level viruses are underrepresented, reinforcing the current need for structural determination focused on potentially (re) emergent viral agents.

## Methods

On September 10th, 2022, the RCSB Protein Data Bank (rcsb.org) (Berman et al. 2000; Burley et al. 2021) was queried for viral protein structures, using the “Browse by Annotations” option under the Search tab. By limiting results by Source Organism as “Viruses”, 10,239 entries were located and a tabular report was generated via databank interface. Custom Python scripts (Supplementary File 1) were developed for extracting information under analysis in this work (number of deposited files per virus, structural determination method employed, average resolution, date of deposition) and for graphically representing the obtained results (as amount of deposited structures by year).

## Results and discussion

The original tabular report obtained from RCSB PDB is shown in Supplementary File 2. It corresponds to 0.18% of the entire database (5,717,483 total). Since we decided to focus on the WHO priority list of human viruses, the original set containing all 10,239 virus entries was filtered, leading to 5662 entries. The taxonomy definitions for each virus are listed on Supplementary File 3. As can be seen on Table 1, the results are dominated by SARS-CoV-2 and HIV 1 depositions. It is possible to observe that Cryo-EM became the second most prevalent structural determination technique, especially in the case of SARS-CoV-2 where it far exceeds NMR in number of structures elucidated. Note that 23 deposited entries were obtained by other methods than X-ray diffraction, nuclear magnetic resonance spectroscopy, or Cryo-EM, and are not represented in this list.

**Table 1:** Structural entries for the WHO priority list of viruses

Virus	X-Ray	Cryo-EM	NMR	Avg XR res.	Avg EM res.	Total
SARS-CoV-2	1688	916	13	1.86	3.42	2626
HIV 1	1811	242	115	2.23	4.82	2182
SARS Virus	189	32	28	2.12	3.72	249
Zika virus	141	22	2	2.11	7.41	165
MERS virus	123	22	0	2.30	3.77	145
Ebola virus	67	22	1	2.39	4.24	90
Lassa fever virus	30	18	1	2.31	4.09	49
HIV 2	26	0	8	2.15	–	34
Rift Valley fever virus	22	6	1	2.51	10.79	29
Nipah virus	20	6	0	2.44	3.48	26
Henipa virus	25	1	0	2.63	2.80	26
Crimean-Congo hemorrhagic fever virus	20	1	1	2.27	2.80	22
Marburg virus	18	1	0	2.48	3.10	19
						5662

*X-Ray* X-ray diffraction, *Cryo-EM* cryogenic electron microscopy, *NMR* nuclear magnetic resonance spectroscopy, *Avg XR res.* average x-ray resolution (Å), *Avg EM res.* average cryo-EM resolution (Å)

**Fig. 1:**
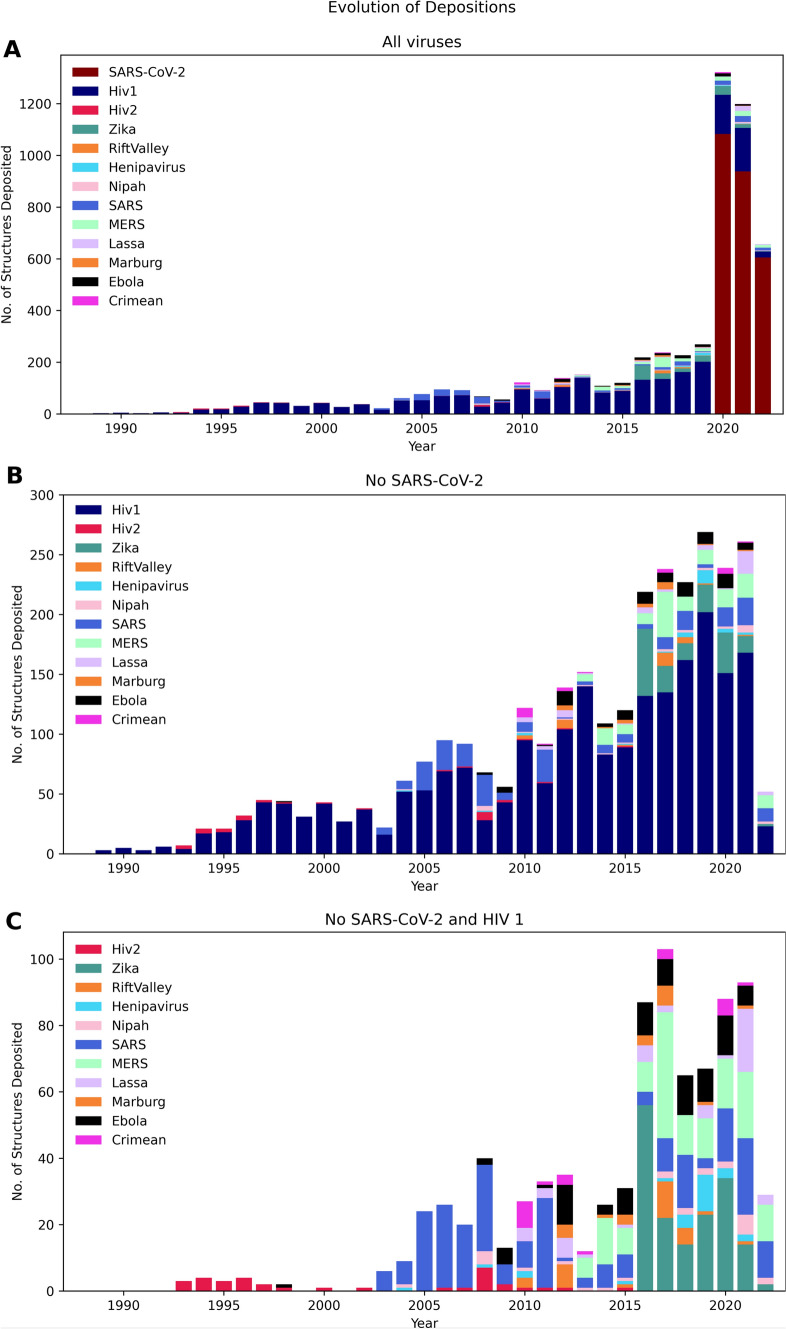
Time-evolution of the viral structures deposited in the PDB for the selected viruses in this work. (Note the difference in scale; see text for details)

In order to inspect potential trends in viral proteins structural determination, we plotted the number of structures deposited per virus in the databank by year (Fig. 1A). As expected, the number of structures from the SARS-CoV-2 virus has increased drastically in the last three years, due to the efforts in studying the virus during the COVID-19 pandemic. Values reached more than a thousand structures in just about 1 year (2020) and were kept up above 500 structures in subsequent years. When we remove SARS-CoV-2 structure entries from the plot (Fig. 1B), the number of structures obtained falls sharply to a maximum of 200 entries per year. A clear dominance can be observed in this case for HIV 1, followed by minor spikes in structures from other viruses, namely SARS-CoV-1, MERS, and Zika. Further removal of HIV 1 structures from the plot (Fig. 1C) reduces the number of annual entries to less than 50 entries annually, with the exception of 2016 following the Zika epidemic of 2015/2016. In the absence of SARS-CoV-2 and HIV 1, most structures deposited are from SARS-CoV-1, MERS, Zika, Ebola, LASSA and Crimean virus. Altogether, in the context of the WHO priority list of diseases, the analyzed data indicates that there is a lack of species diversity in the so far obtained viral protein structures. This observation, nonetheless, must be taken considering the timeframe since the establishment of the WHO priority list, originally proposed in 2017 in response to a previous Ebola virus major outbreak. Thus, not enough time might have passed to ensure viral diversity in the PDB depositions. Simultaneously, efforts were made to supply the demand for specific infectious agents in times of crisis, especially SARS-CoV-2. The advances in tools and methodologies (Stollar and Smith 2020), particularly in cryo-EM, along with advances in beamlines, diffractometers and detectors, also account for the outstanding increase in structure depositions during the SARS-COV-2 pandemic. The data presented here might also overlook deposited complexes that include viral peptides and non-viral binders, considering the taxonomy-based approach used to obtain the structural records.

Considering that structure-based drug design (SBBD) or rational drug design are important fields in the discovery of new drug candidates, obtaining novel structures is an essential step for antiviral development. Diseases with epidemic potential like Rift Valley Fever, Henipah/Nipah and Marburg are far behind in terms of available structures to perform such studies, despite not requiring the highest biosafety level for cloning and expressing the proteins of interest. One way to alleviate this issue is to make use of computational methods (e.g. comparative modeling, fold recognition, de novo modeling) or tools like AlphaFold 2 (Jumper et al. 2021) to provide theoretical solutions to the unknown protein structures. These models are frequently much faster to obtain and of acceptable quality. One example is described in a recent report (Narykov et al. 2021) which has shown that computational models of SARS-CoV-2 proteins produced using a combination of comparative modeling and de novo modeling achieved reasonably accurate structures (average root mean squared deviation error of 4.1 Å), while covering 80% of the viral protein sequence (vs 82% from experimental structures). On average, the computational structures were obtained 86 days earlier than the experimental ones. This shows the potential of computational methods in accelerating SBDD projects, especially with novel AI-powered prediction techniques (such as AlphaFold 2) being pushed forward. Despite that, experimentally obtained structures are still of utmost importance, especially when studying the diversity of multiprotein complexes which are commonly formed by viral proteins (Kuhlman and Bradley 2019) and which are harder to predict when using only computational methods. Likewise, AI-based prediction techniques might need additional data to fully encompass the diversity of viral protein structure repertoire (Narykov et al. 2021).

Viral diseases have caused a major impact in human life. Smallpox and HIV are good examples of viruses that have taken the life of millions and millions of people (Nathanson 2016). New viral pandemics or epidemics are expected to occur in the future, possibly from newly emerging or re-emerging viral diseases. For example, if we take the three viruses in our work with the least structures resolved in the PDB, namely Marburg virus, Crimean-Congo hemorrhagic fever virus and Nipah/Henipah virus, we have diseases with, respectively, 24–88%, 40%, and 40–70% case fatality rates. Such high mortality rates are alarming and highlight the importance of better understanding these viral diseases. Current forecasts indicate that deforestation, climate change and the viral diversity in itself are all concurring to promote more frequent viral spillovers of pandemic proportions (Carlson et al. 2022; Pandit et al. 2022). From this perspective, in this report, we demonstrate that there is a need to increase the efforts in viral structures determination in order to increase preparedness for future challenges.

## Supplementary Information

Below is the link to the electronic supplementary material.

Supplementary file1 (TXT 4 KB)

Supplementary file2 (CSV 1960 KB)

Supplementary file3 (XLSX 10 KB)

Supplementary file4 (PY 4 KB)

## Data Availability

All data pertaining to this article is available in the Supplementary Information.
